# Ssri-treated psychiatric disorders prediction with AI

**DOI:** 10.1192/j.eurpsy.2021.1305

**Published:** 2021-08-13

**Authors:** A. Mereu

**Affiliations:** Research performed independently, Cagliari, Italy

**Keywords:** mood disorders, anxiety disorders, Artificial Intelligence, serotonin

## Abstract

**Introduction:**

SSRI-treated psychiatric disorders (STPD), such as general anxiety disorder and major depression disorder, are common psychiatric diagnoses. Serotonin-mediated effects of solar insolation are an active topic of research. Artificial intelligence (AI) could help to better examine that complex relation.

**Objectives:**

To investigate whether AI could predict the STPD relying primarily on average ambient temperature and annual solar insolation.

**Methods:**

Data of age, average ambient temperature and annual solar insolation were employed to predict STPD status in 7,587 subjects using an AI. To simplify the data analysis, only individuals with white ethnicity were assessed. SPTD prevalence was 17.1%. The AI was conservatively tuned to maximize the positive likelihood ratio considering predicted and real STPD statuses. The free and open source programming language R was used for all the analyses. Dataset source: Wortzel, Joshua; Kent, Shia; Avery, David; Al-Hamdan, Mohammad; Turner, Brandon; Norden, Justin; Norden, Michael; Haynor, David (2018), “Data for: Ambient temperature and solar insolation are associated with decreased prevalence of SSRI-treated psychiatric disorders”, Mendeley Data, V1, doi: 10.17632/trs43ybh92.1

**Results:**

Predictions obtained a positive likelihood ratio of 4.850. The results were indicative of fair performance.

**Conclusions:**

AI might be useful to predict STPD. Furthermore, the results of this study might indicate a moderate effect of age, average ambient temperature and annual solar insolation on the probability of STPD occurrence. Finally, the AI used in this study is freely available, allowing anyone to experiment.

